# Total Flavonoids from* Oroxylum indicum* Induce Apoptosis via PI3K/Akt/PTEN Signaling Pathway in Liver Cancer

**DOI:** 10.1155/2018/3021476

**Published:** 2018-02-15

**Authors:** Nan-nan Li, Xian-sheng Meng, Wen-xiao Men, Yong-rui Bao, Shuai Wang

**Affiliations:** ^1^School of Pharmacy, Liaoning University of Traditional Chinese Medicine, Dalian 116600, China; ^2^Component Medicine Engineering Research Center of Liaoning Province, Dalian 116600, China; ^3^Liaoning Province Modern Chinese Medicine Research Engineering Laboratory, Dalian 116600, China

## Abstract

Total flavonoids (TF), derived from the seeds of* Oroxylum indicum *(L.) Vent., possess many pharmacological functions. In the present study, H_22_-bearing mice and SMMC-7721 models were employed to evaluate the antitumor activity of TF and to and investigate its possible mechanisms both* in vitro* and* in vivo*. Cell viability was evaluated by MTT assay; cell apoptosis rate was analyzed via Annexin V-FITC/PI double staining by flow cytometer. Meanwhile, the expressions of apoptosis-related mRNA and proteins were evaluated by RT-PCR and Western blot analysis. The results revealed that TF could significantly inhibit the tumor growth, and the possible mechanism was related to the effect of inducing tumor cells apoptosis through PI3K/Akt/PTEN signaling pathway. This study has provided a theoretical basis for the further development and application of TF as antitumor drugs.

## 1. Introduction

Liver cancer, also known as hepatocellular carcinoma (HCC), and fewer special cases, which could include hepatoblastoma and angiosarcoma [1], is the 6th most common type of cancer in the world, with over 700,000 newly diagnosed patients per year. This burden of hepatocellular carcinoma will keep increasing till 2030 [2]. Moreover, smoking, alcohol abuse, and a history of diabetes mellitus may exacerbate the development of HCC [3]. Fluorouracil and tegafur are common, but not satisfactory, treatment of liver cancer, since they may lead to critical side effects and drug resistance [4]. A more effective therapy at present for liver cancer is transplantation, which, unfortunately, possesses a higher chance of metastasis and recurrence for such surgical resection [5]. Despite lack of scientific verification Traditional Chinese medicine (TCM) has been extensively used to treat different kinds of diseases due to its low toxicity, no drug resistance, and high efficiency [6, 7]. Therefore the development of active ingredients derived from TCM, which possess better effectiveness and lower toxicity, has become an urgently necessary study for improving the treatment of liver cancer.

*Oroxylum indicum *(L.) Vent. has been extensively applied to treat cough, pertussis, pharyngitis, acute or chronic bronchitis, and other respiratory disorders. Current studies showed that TF are the main active components of* Oroxylum indicum *(L.) Vent. [8], which mainly contains oroxylin A, oroxylin B, chrysin, chrysin-7-O-*β*-D glycosides, baicalein, and so on [9], with many pharmacological functions such as antiallergic [10], antioxidant [11], antimicrobial [12], anti-inflammatory [13], and antitumor [14] functions. However, investigations on the antihepatoma effects of TF are limited, and the possible molecular mechanisms of the antitumor effects are still unrevealed. The present study was therefore aimed at investigating antitumor effect of TF and its possible molecular mechanisms.

## 2. Materials and Methods

### 2.1. Isolation and Purification of the TF

*Oroxylum indicum* was purchased from Tongrentang Pharmacy and authenticated by Professor Yanjun Zhai (School of Pharmacy, Liaoning University of Traditional Chinese Medicine, Liaoning, China) as dry seeds of* Oroxylum indicum *(L.) Vent. Using the best extraction and purification process which was got from the early research of the laboratory,* Oroxylum indicum* was extracted three times with 15 times the amount of 60% ethanol for 1 h. HPD-100 resin was used, with the sample concentration of 0.1 g·mL^−1^, and HPD-100 resin with 5 : 1 of crude drugs BV column, after sufficient adsorption, with 5 BV water to remove impurities and 10 BV 60% ethanol elution, named TF, purity ≥ 90%.

### 2.2. Tumor Cell Lines

Mouse H_22_ hepatocarcinoma cells (H_22_) were obtained from the Shanghai Cell Bank, Chinese Academy of Science (Shanghai, China), and maintained in the peritoneal cavity of male ICR mice, used for further experiments.

### 2.3. Animals and Sample Preparation

Adult male ICR mice weighing 18–22 g were purchased from the experimental animal centre of Liaoning Changsheng Biological Technology Co., Ltd. Mice (except control group) were inoculated with 0.25 ml of 5 × 10^5^/mL H_22_ cell suspension in the right armpit subcutaneously [15, 16]. The animals were randomly divided into six groups, each consisting of twelve mice including blank group, control group, low-dose TF (TFL 50 mg/kg), medium-dose TF (TFM 100 mg/kg), and high-dose TF (TFH 200 mg/kg) groups, and cyclophosphamide positive group (50 mg/kg). On the second day after the inoculation, the mice expect control and model groups were given the different doses of TF and cyclophosphamide intragastrically (blank and model control groups were given the same volume of normal saline) once daily consecutively for 12 d; members of our research team observed mice daily for clinical signs of illness (e.g., weight loss and inactivity). Then mice were prohibited from any food but were allowed access to water freely for 12 h before the experiments; on the 12th day morning, taking drugs 1 h later, the body weight of mice was measured and the blood was collected by removing eyeball under sodium pentobarbital anesthesia (1% sodium pentobarbital, 50 mg/kg, intraperitoneal injection) in order to alleviate animal suffering. For collection of mouse tissues, mice were euthanized by carbon dioxide inhalation, and death confirmed by ascertaining cardiac and respiratory arrest. The tumors were removed and weighed; the tumor inhibition rate was calculated by the following formula: tumor inhibition rate = (1 − the average tumor weight medicine group/the average tumor weight control group) × 100% [17, 18].

### 2.4. Histopathological Examination

For the histopathological analysis of tumor by HE straining method, tumor tissues of each group were fixed in 10% formalin solution for over 24 h. After routine processing, the tumors were embedded in paraffin and cut at a thickness of 5*μ*m. Sections were stained with hematoxylin and eosin (H&E) and subsequently examined using a light microscope for histopathological examination.

### 2.5. Measurement of BAX and Bcl-2 Level by ELISA Assay

The ELISA assay was performed to quantify tumor tissue levels of BAX and Bcl-2 according to the manufacturer's instructions. The absorbance was measured with a Spectra Max Plus microplate reader (Molecular Devices, CA, USA) at a wavelength of 450 nm.

### 2.6. Cell Culture

The human hepatoma cell line SMMC-7721 was purchased from the Shanghai Cell Bank, Chinese Academy of Science (Shanghai, China). This cell line was cultured in DMEM medium (Gibco, Grand Island, USA) supplemented with 1% penicillin/streptomycin (Gibco, Grand Island, USA) and 10% FBS (fetal bovine serum) (Gibco, Grand Island, USA) at 37°C in a humidified incubator containing 5% CO_2_.

### 2.7. MTT Colorimetric Assay

MTT assay was used to determine cell viability. Briefly, cells were plated in 96-well plates at a density of 1 × 10^4^ per well. After overnight culture, different concentrations of TF (0.2, 0.6, and 1.0 mg/mL) were added to the wells and cells were incubated for 24, 48, and 72 h. Then the culture medium was replaced with MTT (Sigma St. Louis, MO, USA) at final concentration of 2.5 mg/mL, followed by 4 h incubation. Additionally, 150*μ*L of Dimethyl sulfoxide (DMSO) (Sigma St. Louis, MO, USA) was added to each well. Absorbance was measured with a Spectra Max Plus microplate reader (Molecular Devices, CA, USA) at a wavelength of 492 nm.

### 2.8. Cell Apoptosis

The apoptosis analyses by flow cytometer used FITC/PI double staining method; TF group cells including treated cells (cells were treated with TF for 48 h) were collected and rinsed twice with cold PBS, mixed with 500*μ*L of 1x binding buffer (1 × 10^6^/mL), 5*μ*L Annexin V-FITC, and 5*μ*L propidium iodide and incubated in the dark for 15 min, and finally sent to the BD Accuir C6 flow cytometer (BD, USA) to analyze the cell apoptosis.

### 2.9. RNA Isolation and Real-Time Quantitative PCR Analysis

The human hepatoma cell line SMMC-7721 was plated in 6-well plates at a density of 5 × 10^4^ per well. After overnight culture, the TF at concentration of 1.0 mg/mL was added to the wells and cells were incubated for 48 h. Total RNA of each group cells was extracted using TRIzol (Ambion, Texas, USA) according to the manufacturer's recommendations. Total RNA was reverse-transcribed using the TransScript First-Strand cDNA Synthesis SuperMix (TransGen Biotech, China). Quantitative real-time PCR was performed using TransStart Top Green Qpcr SuperMix (TransGen Biotech, China) with Piko Thermal Cycler 96-well system (Piko, Hawaii State, USA). Each sample was analyzed in triplicate. Relative levels of mRNA expression were normalized for*β*-actin mRNA expression and calculated according to the formula 2^−(ΔCt  sample−ΔCt  control)^. Primers used were listed in Table 1. All primers were synthesized by Invitrogen, USA.

### 2.10. Western Blotting Analysis

The human hepatoma cell line SMMC-7721 was plated in 6-well plates at a density of 5 × 10^4^ per well. After overnight culture, the TF at concentration of 1.0 mg/mL was added to the wells and cells were incubated for 48 h. Protein extracts were isolated from each group cells in using RIPA protein lysis buffer containing 1 mM PMSF. Total protein was separated by 10% SDS-PAGE, transferred with polyvinylidenedifluoride (PVDF) membrane, blocked in 5% BSA (Solarbio, Beijing, China), and probed with appropriate primary antibodies against the target proteins. PTEN, PI3 Kinase p85, p-Akt, and*β*-actin antibodies were purchased from Cell Signaling (Beverly, MA, USA). These were followed by incubation with Goat Anti-Rabbit immunoglobulin (Ig) G (H + L) (Protein tech, USA), and antigen-antibody complexes were visualized using the chemilucent ECL (TransGen Biotech, China) detection system. The densitometric analysis was conducted by using custom Image J-based software, as reported earlier [19, 20].

### 2.11. Statistics Analysis

Statistical analysis was performed using *t*-test or one-way ANOVA with GraphPad Prism 5. The *p* values were considered statistically significant at *p* < 0.05, and *p* < 0.01 for very significant difference. All data are means ± standard deviation (SD) for at least three separate experiments.

## 3. Results

### 3.1. Effect of TF on Tumor Growth in H_22_ Tumor-Bearing Mice

**Figure 1:**
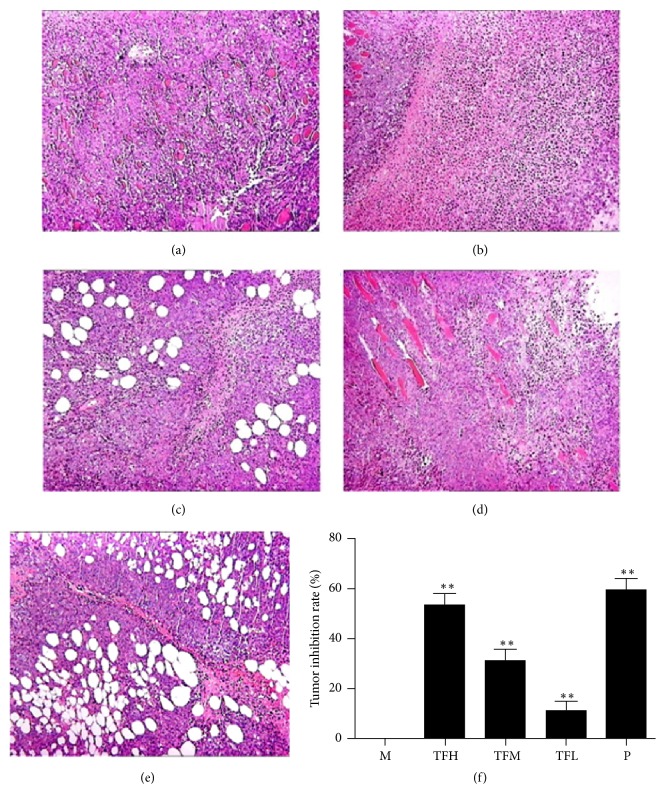
The effect of TF on tumor growth of H_22_ tumor-bearing mice. (a) Histopathological examination of control group, (b) positive group, (c) high-dose group, (d) medium-dose group, and (e) low-dose group. These tissues are stained by H&E (200x). (f) The results of tumor inhibition rate, ^*∗∗*^*p* < 0.01.

The histopathological examination was in order to evaluate the antitumor effect of TF; as shown in Figures 1(a)–1(e), the model group had large areas and density of tumor cells, which indicated that H_22_-bearing model was successfully established. The mice in positive and low-, medium-, and high-dose TF groups showed different degree of apoptosis areas compared to the model group.

The antitumor effect of TF on H_22_ tumor-bearing mice was showed in Figure 1(f). The results indicated that after the administration of TF for 12 d the average tumor weight was significantly decreased (*p* < 0.01 or *p* < 0.05) compared to the model group.

### 3.2. Effect of TF on Tumor Tissue Levels of BAX and Bcl-2 in H_22_ Tumor-Bearing Mice

**Figure 2:**
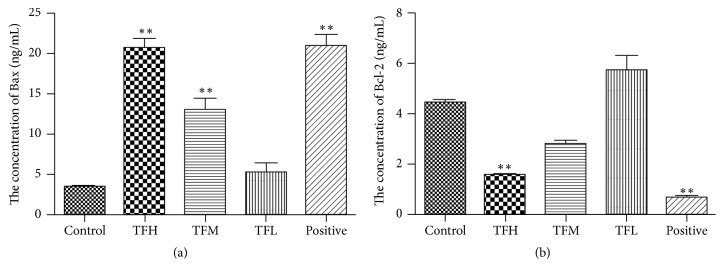
The levels of Bax and Bcl-2 in tumor tissue of H_22_ tumor-bearing mice. (a) The concentration of Bax. (b) The concentration of Bcl-2. All data were expressed as mean ± SD, *n* = 6. ^*∗∗*^*p* < 0.01.

We used the ELISA assay to investigate the effects of TF on the levels of BAX and Bcl-2 in tumor tissues of H_22_ tumor-bearing mice. As shown in Figures 2(a)-2(b), the results indicated that the levels of BAX were significantly increased in the mice in medium- and high-dose (*p* < 0.01) compared with the control group, and Bcl-2 level of the mice in high-dose TF groups was significantly decreased when compared with the control group (*p* < 0.01).

### 3.3. Effect of TF on the Proliferation in the Human Hepatoma Cell Line SMMC-7721

**Figure 3:**
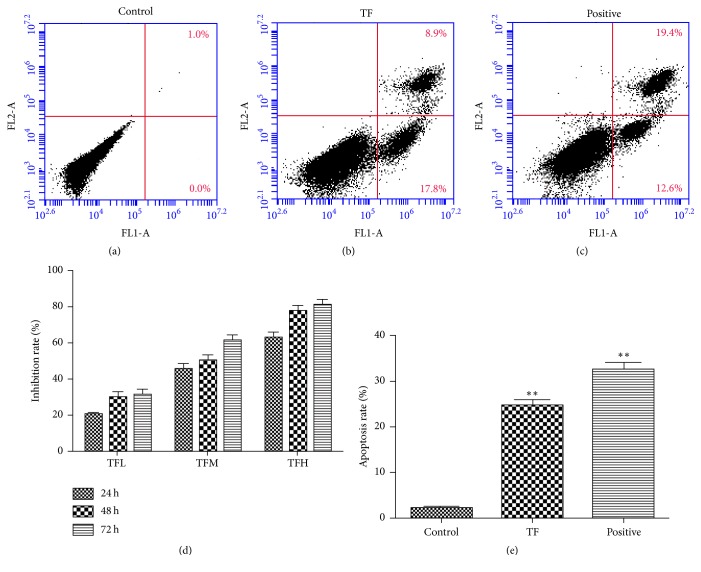
The effect of TF on apoptosis of SMMC-7721 cells. (a) Control group, (b) TF group, (c) positive group, and (e) statistical analysis results of cell apoptosis ratio by flow cytometry. ^*∗∗*^*p* < 0.01 versus control group; (d) TF inhibits the proliferation in the human hepatoma cell line SMMC-7721. Inhibition ratio of SMMC-7721 cells by TF with different concentrations (0.2, 0.6, and 1.0 mg/mL) for 24, 48, and 72 h. All data were expressed as mean ± SD, *n* = 6.

In order to investigate the effect of TF on the proliferation of human hepatoma cell line SMMC-7721, we set up different concentrations of TF (0.2, 0.6, and 1.0 mg/mL) and then incubated cells for 24, 48, and 72 h. The results indicated that TF could inhibit the proliferation of SMMC-7721 cells in a dose-dependent manner (Figure 3(d)).

### 3.4. Effect of TF on Apoptosis in the Human Hepatoma Cell Line SMMC-7721

**Figure 4:**
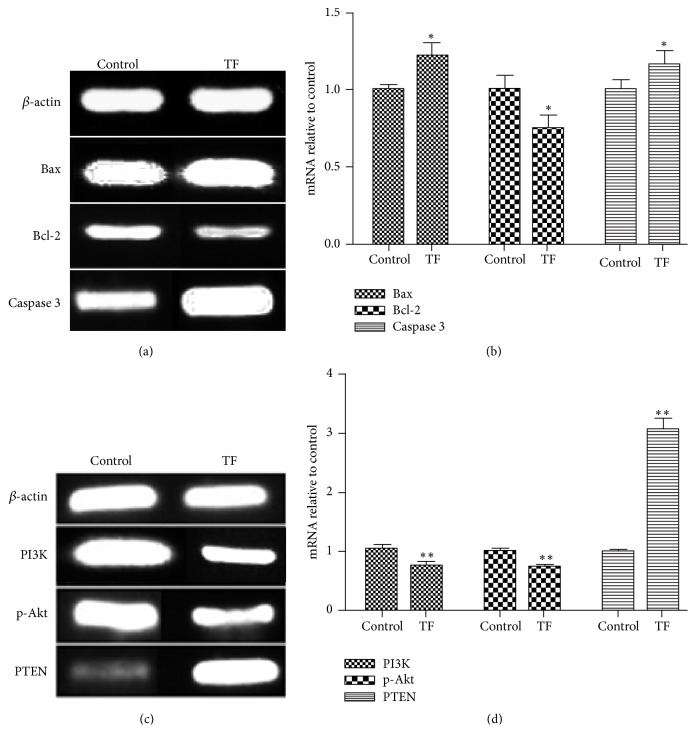
The expression of Bax, Bcl-2, Caspase 3, p-Akt, PI3K, and PTEN mRNA in SMMC-7721 cells. (a) The expression of Bax, Bcl-2, and Caspase 3 mRNA in SMMC-7721 cells and (b) Bax, Bcl-2, and Caspase 3 mRNA relative to control; (c) the expression of p-Akt, PI3K, and PTEN mRNA in SMMC-7721 cells and (d) PI3K, p-Akt, and PTEN mRNA relative to control. The levels of mRNAs were detected by RT-PCR and measured with *β*-actin as an internal reference. All data, repeated by three independent experiments, are presented as mean ± SD. ^*∗*^*p* < 0.05 and ^*∗∗*^*p* < 0.01 versus control group.

The flow cytometry analysis showed that the percentage of apoptosis in TF group was 24.77 ± 1.90%; TF could significantly promote cell apoptosis when compared with the control group (*p* < 0.01), as shown in Figures 3(a)–3(c) and 3(e). We also determined the apoptosis of SMMC-7721 cell lines by RT-PCR analyses of Bax, Bcl-2, and Caspase 3. The results showed that the expression of Bcl-2 was significantly decreased, and Bax and Caspase 3 were increased versus control group (^*∗∗*^*p* < 0.01, ^*∗*^*p* < 0.05) after cells were treated by TF (Figures 4(a)-4(b)). To summarize, these results suggested that TF inhibited cell proliferation by apoptosis.

### 3.5. The Effect of TF on p-Akt, PI3K, and PTEN mRNA Expression in SMMC-7721 Cells

To investigate the effect of TF on p-Akt, PI3K, and PTEN expression in SMMC-7721 cells, we detected the expression by two-step RT-PCR analysis. As shown in Figures 4(c)-4(d), the expression of p-Akt and PI3K was significantly decreased, and PTEN increased versus control group (^*∗∗*^*p* < 0.01, ^*∗*^*p* < 0.05) after being treated by TF.

### 3.6. The Effect of TF on PI3K, p-Akt, and PTEN Protein Expression in SMMC-7721 Cells

**Figure 5:**
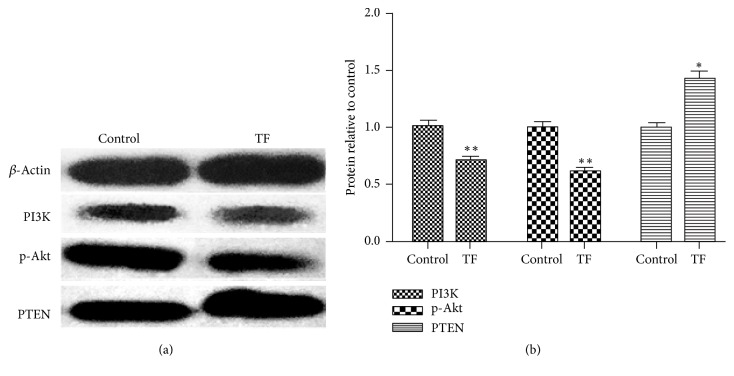
The expression of p-Akt, PI3K, and PTEN protein in SMMC-7721 cells. (a) Protein expression levels of PI3K, p-Akt, and PTEN in SMMC-7721 cells following TF treatment for 48 h. (b) The expression of PI3K, p-Akt, and PTEN protein relative to control. All data, repeated by three independent experiments, are presented as mean ± SD. ^*∗*^*p* < 0.05 and ^*∗∗*^*p* < 0.01 versus control group.

Western blot analyses further confirmed whether lower levels of PTEN, p-Akt, and PI3K protein and higher levels of PTEN were observed in the TF treated cells. As shown in Figures 5(a)-5(b), p-Akt and PI3K protein expression were lower in TF treated cells versus control group (^*∗*^*p* < 0.05 or ^*∗∗*^*p* < 0.01). As shown in Figure 5, the expression of PTEN protein is significantly higher in TF treated cells versus the control group (^*∗∗*^*p* < 0.01).

## 4. Discussion

TF, as the active ingredients of* Oroxylum indicum *(L.) Vent., exert multiple physiological activities, including antitumor, antioxidation, antifatigue, anti-inflammation, and antimicrobial effect. In this study, the antitumor effect of TF and the possible molecular mechanism were investigated both* in vitro* and* in vivo*. From the results of histopathological examination (Figures 1(a)–1(e)), the high-, medium-, and low-dose TF group showed apoptosis in various degree versus control group which demonstrated that TF had good effect on inhibiting tumor cell proliferation and promoting their apoptosis. In addition, TF showed inhibitory effect on tumor growth in H_22_ tumor-bearing mice and SMMC-7721 cells, which exerted significant inhibition in a dose-dependent manner (Figures 1(f) and 3(d)). Result showed that TF had a good antihepatocellular carcinoma effect both* in vitro* and* in vivo*, which also indicated that TF, as a potential antitumor agent, had the value of further research.

Apoptosis, a programmed cell death, is one of the main mechanisms of cell death in tumor treatment [21, 22]. It is regulated by many genes, including caspase and Bcl-2 family genes such as Bax and Bcl-2 [23–25]. The Bax gene is a tumor suppressor gene, and Bcl-2 is an antiapoptotic gene that antagonizes the function of Bax [26, 27]. Moreover, Caspase-3 is the main process in the induction of apoptosis, since it is activated to regulate the caspase-signaling cascade and a downstream effector of apoptosis pathways [28]. In this study, the results of HE straining and flow cytometry showed that apoptosis occurred both in tumor tissues and in SMMC-7721 cells after TF intervention. We also determined the apoptosis by ELISA assay and RT-PCR method of BAX, Bcl-2, and Caspase 3 levels in tumor tissues of H_22_ tumor-bearing mice and SMMC-7721 cells. These results suggested that, in either tumor tissues or SMMC-7721 cells, TF could downregulate Bcl-2 and upregulate Bax and Caspase 3 expressions. Therefore, the mechanism of antihepatocellular carcinoma of TF is related to induce apoptosis.

**Figure 6:**
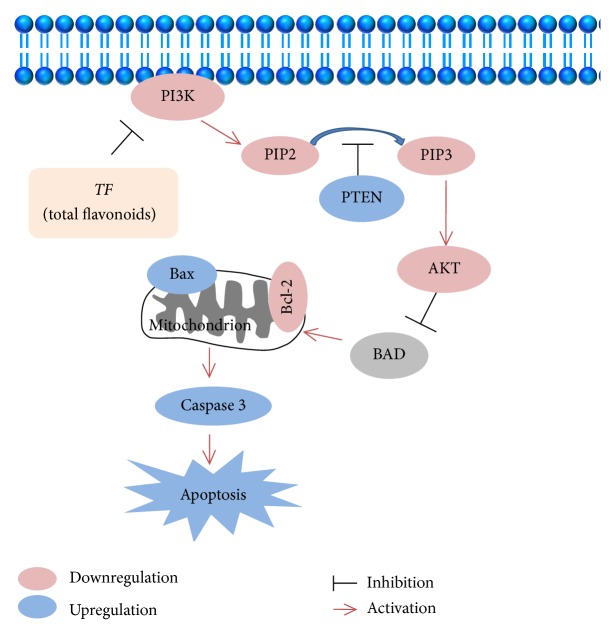
Anti-liver cancer mechanism of TF.

The PI3K/Akt/PTEN signaling pathways are crucial in many physiological and pathological conditions, such as cell proliferation, angiogenesis, and apoptosis [29, 30]. Previous studies showed that PI3K/Akt was frequently improperly activated in many kinds of human cancers [31], including hepatocellular carcinoma. It is closely associated with inhibiting the apoptotic cells and promoted the proliferation [32]. The PTEN is a key molecule downstream of the PI3K/Akt pathway and acts as a tumor suppressor by inhibiting cell growth and enhancing apoptosis [33, 34]. In order to verify whether the antitumor effect of TF is related to the PI3K/Akt/PTEN pathway, and how to regulate related targets, PT-PCR and Western blot method were used. From the results of the study (Figures 4-5), TF could downregulate the expression of PI3K, p-Akt mRNA, and protein and upregulate the expression of PTEN. The results indicate that TF inhibited the PI3K/Akt/PTEN pathway and contributed to cellular apoptosis (as shown in Figure 6). In summary, TF acted as antitumor agent against liver cancer by promoting apoptosis via PI3K/Akt/PTEN signaling pathway.

## 5. Conclusion

In conclusion, this research offered evidence that TF could significantly inhibit the growth of tumor* in vitro *and* in vivo*, possibly via inducing tumor cells apoptosis by PI3K/Akt/PTEN signaling pathway. Our research provided a scientific basis for the exploitation and application of total flavonoids in* Oroxylum indicum *(L.) Vent. as an antitumor agent, although further research will be needed before the clinical use.

## Figures and Tables

**Table 1:** RT-PCR primers for each gene.

Gene	Forward primers	Reverse primers
*β*-Actin	5′–CACCCGCGAGTACAACCTTC-3′	5′-CCCATACCCACCATCACACC-3′
Bax	5′-TCATGGGCTGGACATTGGAC-3′	5′-GAGACAGGGACACAGTCGC-3′
Bcl-2	5′-GTGAAGTCAACATGCCTGCC-3′	5′-ACAGCCTGCAGCTTTGTTTC-3′
Caspase 3	5′-CCTGGTTCATCCAGTCGCTT-3′	5′-TCTGTTGCCACCTTTCGGTT-3′
PI3K	5′-AACGAGAACGTGTGCCATTTG-3′	5′-AGAGATTGGCATGCTGTCGAA-3′
p-Akt	5′-CGAGGAGGAGGTGTATCA-3′	5′-CGGTAAAGGCACGTTCGGTA-3′
PTEN	5′-CCCAGTCAGAGGCGCTATG-3′	5′-GGCAGACCACAAACTGAGGATT-3′
